# Arthritis Prevalence Among Veterans — United States, 2017–2021

**DOI:** 10.15585/mmwr.mm7245a1

**Published:** 2023-11-10

**Authors:** Elizabeth A. Fallon, Michael A. Boring, Anika L. Foster, Ellen W. Stowe, Tyler D. Lites, Kelli D. Allen

**Affiliations:** ^1^Division of Population Health, National Center for Chronic Disease Prevention and Health Promotion, CDC; ^2^ASRT, Inc., Smyrna, Georgia; ^3^Oak Ridge Institution for Science and Education, Oak Ridge, Tennessee; ^4^Thurston Arthritis Research Center, Department of Medicine, University of North Carolina at Chapel Hill, Chapel Hill, North Carolina; ^5^Health Services Research & Development, Department of Veterans Administration Health Care System, Durham, North Carolina.

**Summary**

**What is already known about this topic?**

Arthritis is a chronic inflammatory condition that can lead to chronic pain and disability. U.S. veterans experience higher rates of diagnosed arthritis than nonveterans.

**What is added by this report?**

Approximately one third of U.S. veterans reported diagnosed arthritis during 2017–2021. Among men aged 18–44 years, the arthritis prevalence among veterans was double that among nonveterans, and among women aged 18–44 years, the arthritis prevalence among veterans was 60% higher than among nonveterans.

**What are the implications for public health practice?**

Multisectoral partnerships among public health departments, community-based organizations, veteran-serving organizations, health care providers, and payors can help achieve equitable access to arthritis-appropriate, evidence-based programs for veterans to prevent or limit progression of arthritis, particularly among disproportionately affected groups and relatively younger veterans who might have been living longer with arthritis.

## Abstract

Arthritis is a chronic inflammatory condition and a leading cause of chronic pain and disability. Because arthritis prevalence is higher among U.S. military veterans (veterans), and because the veteran population has become more sexually, racially, ethnically, and geographically diverse, updated arthritis prevalence estimates are needed. CDC analyzed pooled 2017–2021 Behavioral Risk Factor Surveillance System data to estimate the prevalence of diagnosed arthritis among veterans and nonveterans, stratified by sex and selected demographic characteristics. Approximately one third of veterans had diagnosed arthritis (unadjusted prevalence = 34.7% [men] and 31.9% [women]). Among men aged 18–44 years, arthritis prevalence among veterans was double that of nonveterans (prevalence ratio [PR] = 2.1; 95% CI = 1.9–2.2), and among men aged 45–64 years, arthritis prevalence among veterans was 30% higher than that among nonveterans (PR = 1.3; 95% CI = 1.3–1.4). Among women aged 18–44 years, arthritis prevalence among veterans was 60% higher than that among nonveterans (PR = 1.6; 95% CI = 1.4–1.7); among women aged 45–64 years, arthritis prevalence among veterans was 20% higher than that among nonveterans (PR = 1.2; 95% CI = 1.1–1.3). Cultivating partnerships with veteran-serving organizations to promote or deliver arthritis-appropriate interventions might be advantageous, especially for states where arthritis prevalence among veterans is highest. The high prevalence of arthritis among female veterans, veterans aged ≥65 years, and veterans with disabilities highlights the importance of ensuring equitable access and inclusion when offering arthritis-appropriate interventions.

## Introduction

Arthritis is a chronic inflammatory condition and a leading cause of chronic pain and disability (*1*,*2*). A recent report determined that arthritis prevalence is higher among U.S. military veterans (veterans) than among nonveterans; 35.2% of veterans (6.8 million) report diagnosed arthritis (*3*). Previous estimates indicate that arthritis prevalence is higher among female veterans, veterans self-identifying as non-Hispanic Black or African American or non-Hispanic White, and those living in southern and Appalachian states (*4*). Because arthritis prevalence is higher among veterans and the veteran population has become more sexually, racially, ethnically, and geographically diverse (*5*), a comprehensive understanding of arthritis prevalence estimates among veterans can guide strategic partnership development and equitable resource allocation for delivery of arthritis-appropriate, evidence-based interventions to veterans.

## Methods

### Data Source and Primary Measures

Behavioral Risk Factor Surveillance System (BRFSS) is an annual, state-based, random-digit–dialed telephone survey of the noninstitutionalized U.S. adult population aged ≥18 years in all 50 states, the District of Columbia (DC), and U.S. territories.* Respondents were classified as having arthritis if they answered “yes” to the question, “Has a doctor, nurse, or other health professional ever told you that you had some form of arthritis, rheumatoid arthritis, gout, lupus, or fibromyalgia?” Respondents were classified as veterans if they answered “yes” when asked, “Have you ever served on active duty in the United States Armed Forces, either in the regular military or in a National Guard or military reserve unit?”

### Data Analysis

CDC analyzed pooled BRFSS data for 2017–2021.^†^ The median response rate during the 2017–2021 survey years ranged from 44.0% to 49.9%,^§^ with a total analytic sample size of 2,087,387.^¶^ Crude, age-specific, and age-standardized** prevalences of diagnosed arthritis were estimated overall and by veteran status, sex, and selected sociodemographic,^††^^,^^§§^^,^^¶¶^^,^***^,^^†††^^,^^§§§^ health,^¶¶¶^^,^**** and disability characteristics.^††††^ T-tests were used to assess differences between veterans and nonveterans overall and by subgroup, as well as to test differences in subgroup categories among veterans using a reference group. All differences are statistically significant at α = 0.05. Age-standardized diagnosed arthritis prevalences among male and female veterans and nonveterans were estimated in the 50 states, DC, and U.S. territories. Prevalence ratios (PRs) were calculated by dividing the prevalence of arthritis among veterans by the prevalence of arthritis among nonveterans. SAS (version 9.4; SAS Institute) and SUDAAN (version 11.0; RTI International) were used for analysis to account for complex design and weighting. This activity was reviewed by CDC, deemed not research, and was conducted consistent with applicable federal law and CDC policy.^§§§§^

## Results

Approximately one third of veterans had diagnosed arthritis (unadjusted prevalence = 34.7% [men] and 31.9% [women]). Age-adjusted prevalence was higher among women (30.5%) than among men (25.2%; p<0.001) (Table 1). Among men, age-specific arthritis prevalences were higher among veterans than among nonveterans across all age groups (18–44, 45–64, and ≥65 years; p<0.001); among women, prevalences were higher among veterans than among nonveterans in two age groups (18–44 and 45–64 years; p<0.001). The age-specific arthritis PRs for veterans compared with nonveterans decreased as age group increased; among men aged 18–44 years, arthritis prevalence among veterans (12.7%) was double that of nonveterans (6.2%; PR = 2.1; 95% CI = 1.9–2.2), whereas among men aged 45–64 years, arthritis prevalence among veterans (34.9%) was 30% higher than that among nonveterans (26.2%; PR = 1.3; 95% CI = 1.3–1.4), and among men aged ≥65 years, arthritis prevalence among veterans (47.2%) was 10% higher than that among nonveterans (42.0%; PR = 1.1; 95% CI = 1.1–1.1). A similar pattern was observed among female veterans and nonveterans. Among women aged 18–44 years, arthritis prevalence among veterans (15.1%) was 60% higher than that among nonveterans (9.5%; PR = 1.6; 95% CI = 1.4–1.7); among women aged 45–64 years, arthritis prevalence among veterans (43.0%) was 20% higher than that among nonveterans (35.8%; PR = 1.2; 95% CI = 1.1–1.3); and among women aged ≥65 years, arthritis prevalence (56.4%) was similar to that among nonveterans (56.1%; PR = 1.0; 95% CI = 1.0–1.1).

**TABLE 1:** Crude, age-standardized,* and age-specific prevalence of diagnosed arthritis^†^ among veterans^§^ and nonveterans, stratified by sex and by selected demographic characteristics — Behavioral Risk Factor Surveillance System, United States, 2017–2021

Characteristic	Prevalence, % (95% CI)			
	Men n = 947,180		Women n = 1,139,254	
	Nonveterans	Veterans	Nonveterans	Veterans
Crude	17.7 (17.5–17.9)	34.7 (34.3–35.1)^¶^	28.8 (28.6–29.0)	31.9 (30.8–33.1)**
Age-standardized	18.3 (18.1–18.4)	25.2 (24.8–25.7)^¶^	25.3 (25.2–25.5)	30.5 (29.4–31.5)**
**Age group, yrs**				
18–44	6.2 (6.0–6.3)	12.7 (12.0–13.4)^¶^	9.5 (9.3–9.7)	15.1 (13.8–16.5)**
45–64	26.2 (25.9–26.5)	34.9 (34.1–35.7)^¶^	35.8 (35.5–36.1)	43.0 (41.1–44.8)**
≥65	42.0 (41.4–42.6)	47.2 (46.7–47.8)^¶^	56.1 (55.7–56.5)	56.4 (53.7–59.0)
**Race and ethnicity** **^††^**				
Black or African American	18.5 (17.9–19.0)	25.6 (24.0–27.2)^¶^	26.9 (26.4–27.4)	29.3 (26.9–31.8)
White	20.3 (20.2–20.5)	25.5 (25.0–26.0)^¶^	27.1 (27.0–27.3)	32.7 (31.5–34.0)**
Hispanic or Latino	12.7 (12.2–13.3)	22.1 (20.4–23.9)^¶^	21.1 (20.6–21.6)	28.7 (25.1–32.6)**
Other	14.2 (13.5–14.9)	26.5 (24.6–28.4)^¶^	19.8 (19.1–20.5)	22.6 (19.0–26.8)
**Highest educational attainment^§§^**				
Less than HS graduate	20.2 (19.7–20.7)	32.1 (28.0–36.5)^¶^	29.3 (28.8–29.9)	35.0 (28.0–42.8)
HS graduate or equivalent	19.7 (19.4–20.0)	23.7 (23.0–24.4)^¶^	26.7 (26.4–27.0)	29.1 (26.9–31.4)**
Technical school degree or some college	19.8 (19.5–20.2)	27.3 (26.5–28.1)^¶^	27.4 (27.1–27.7)	33.3 (31.5–35.1)**
College degree or more	14.6 (14.4–14.8)	23.2 (22.4–23.9)^¶^	20.3 (20.1–20.5)	28.2 (26.8–29.6)**
**Annual household income^¶¶^**				
<$15,000	23.4 (22.7–24.1)	33.3 (30.7–35.9)^¶^	34.3 (33.7–34.9)	41.5 (35.5–47.8)**
$15,000 to <$25,000	21.3 (20.8–21.8)	28.6 (27.3–30.0)^¶^	30.5 (30.0–30.9)	35.7 (32.9–38.6)**
$25,000 to <$50,000	18.9 (18.5–19.3)	26.1 (25.2–27.0)^¶^	26.8 (26.5–27.2)	35.2 (33.0–37.4)**
≥$50,000	16.8 (16.6–17.1)	24.2 (23.5–24.8)^¶^	21.8 (21.6–22.0)	27.8 (26.4–29.3)**
**BMI (kg/m^2^** )***				
Underweight/Healthy weight (<25)	14.4 (14.1–14.7)	20.0 (19.2–20.9)^¶^	19.7 (19.4–19.9)	25.0 (23.3–26.7)**
Overweight (25 to <30)	16.7 (16.4–16.9)	23.1 (22.4–23.7)^¶^	24.4 (24.1–24.7)	30.5 (28.7–32.4)**
Obesity I (30 to <35)	21.9 (21.5–22.3)	30.0 (28.9–31.2)**	30.1 (29.7–30.5)	36.6 (33.9–39.3)**
Obesity II (≥35)	28.9 (28.3–29.5)	36.7 (34.9–38.4)**	38.2 (37.8–38.7)	44.2 (40.8–47.7)**
**Health insurance type^†††^**				
Employer- or union-sponsored	16.6 (16.1–17.2)	21.2 (19.9–22.6)	22.1 (21.5–22.6)	23.5 (20.2–27.0)
Medicare and Medigap	26.9 (25.8–28.0)	38.1 (32.4–44.0)^§§§^	37.3 (36.2–38.5)	37.0 (30.7–43.8)^¶¶¶^
Medicaid or other state program	22.7 (21.5–23.9)	27.5 (22.4–33.2)^§§§^	31.2 (30.1–32.3)	39.9 (33.3–46.8)^¶¶¶^
Self-insured (purchased by self or family member)	16.7 (15.7–17.8)	19.7 (17.3–22.3)	22.1 (21.3–22.9)	26.9 (14.9–43.5)
TRICARE (formerly CHAMPUS), VA, or military	20.2 (15.1–26.5)	31.1 (29.6–32.6)^§§§^	27.8 (25.2–30.4)	34.7 (31.4–38.1)^¶¶¶^
Other health insurance	19.1 (17.4–21.0)	26.1 (21.4–31.6)	27.4 (25.6–29.3)	36.6 (26.8–47.7)^¶¶¶^
None	11.1 (9.6–12.7)	22.4 (16.4–29.9)	16.6 (15.0–18.5)	—****
**Sexual orientation^††††,§§§§^**				
Bisexual	19.7 (17.9–21.8)	27.1 (23.3–31.2)^¶^	30.7 (29.3–32.1)	33.5 (26.8–41.0)
Gay or lesbian	19.7 (18.2–21.3)	24.5 (20.2–29.4)	29.4 (27.5–31.4)	31.1 (24.7–38.2)
Straight or heterosexual	18.6 (18.4–18.8)	25.4 (24.7–26.0)^¶^	25.5 (25.2–25.7)	31.4 (29.9–33.0)**
Something else or don't know	14.4 (13.1–15.8)	27.8 (22.5–34.0)^¶^	20.8 (19.6–22.0)	31.6 (24.5–39.7)**
Self-rated health^¶¶¶¶^				
Excellent/Very good	13.0 (12.8–13.2)	17.7 (17.2–18.3)^¶^	17.0 (16.9–17.2)	20.9 (19.8–22.1)**
Good	19.4 (19.1–19.7)	28.3 (27.4–29.2)^¶^	27.2 (26.9–27.5)	34.4 (32.3–36.4)**
Fair/Poor	30.9 (30.4–31.4)	44.7 (42.8–46.6)^¶^	44.7 (44.2–45.2)	55.3 (51.3–59.2)**
Hearing disability*****				
Yes	32.2 (31.2–33.2)	42.4 (40.4–44.4)^¶^	42.2 (41.0–43.4)	51.2 (44.9–57.5)**
No	17.4 (17.2–17.5)	23.2 (22.8–23.7)^¶^	24.7 (24.6–24.9)	29.6 (28.6–30.7)**
Vision disability*****				
Yes	29.6 (28.6–30.6)	40.4 (37.4–43.5)^¶^	40.2 (39.3–41.2)	51.4 (43.8–59.0)**
No	17.8 (17.6–17.9)	24.7 (24.2–25.1)^¶^	24.6 (24.4–24.7)	29.7 (28.7–30.8)**
Cognitive disability*****				
Yes	32.6 (31.9–33.3)	47.2 (45.4–49.0)^¶^	43.6 (43.1–44.2)	55.0 (51.8–58.2)**
No	16.8 (16.6–17.0)	22.1 (21.7–22.6)^¶^	22.7 (22.5–22.9)	26.3 (25.3–27.4)
Mobility disability*****				
Yes	46.6 (45.6–47.6)	58.0 (55.4–60.5)^¶^	61.8 (61.0–62.6)	68.6 (64.4–72.6)**
No	15.1 (14.9–15.2)	20.6 (20.1–21.0)^¶^	20.0 (19.8–20.1)	23.9 (22.9–24.9)**
Self-care disability*****				
Yes	48.0 (46.4–49.6)	63.5 (59.8–67.0)^¶^	65.9 (64.4–67.3)	72.5 (65.0–78.9)
No	17.3 (17.2–17.5)	23.6 (23.2–24.1)^¶^	24.0 (23.9–24.2)	28.8 (27.8–29.8)**
Independent living disability*****				
Yes	39.0 (38.1–40.0)	52.4 (49.8–55.0)^¶^	51.2 (50.4–51.9)	63.7 (59.4–67.8)**
No	17.2 (17.1–17.4)	23.3 (22.9–23.8)^¶^	23.1 (23.0–23.3)	27.3 (26.3–28.4)**

**Abbreviations**: BMI = body mass index; HS = high school; VA = Veterans Health Administration.

* Age-standardized to the 2000 U.S. Census Bureau projected adult population, using three age groups: 18–44, 45–64, and ≥65 years. https://www.cdc.gov/nchs/data/statnt/statnt20.pdf

^†^ Responded “yes” to the question, “Have you ever been told by a doctor or other health professional that you had some form of arthritis, rheumatoid arthritis, gout, lupus, or fibromyalgia?”

^§^ Veterans were defined as respondents who answered “yes” to the question, “Have you ever served on active duty in the United States Armed Forces, either in the regular military or in a National Guard or military reserve unit?”

^¶^ Statistically significant (p≤0.05) difference between male veterans and male nonveterans (reference group).

** Statistically significant (p≤0.05) difference between female veterans and female nonveterans (reference group).

^††^ Persons of Hispanic or Latino (Hispanic) origin might be of any race but are categorized as Hispanic; all racial groups are non-Hispanic. Persons self-identifying as American Indian or Alaska Native, Asian, Native Hawaiian or other Pacific Islander, other race, or multiracial were combined into “Other.”

^§§^ Responses to the question, “What is the highest grade or year of school you completed?” were combined into the following groups: 1) less than HS graduate: never attended school or attended only kindergarten, grades 1–8, or grades 9–11; 2) HS graduate or equivalent: grade 12 or general educational development certificate; 3) technical school degree or some college: college 1–3 years; and 4) college degree or more: college ≥4 years.

^¶¶^ The calculated variables for income were harmonized across years to create four groups. For 2021 data, the following responses were combined into the “≥$50,000” category: $50,000 to <$100,000, $100,000 to <$200,000, and ≥$200,000.

*** The calculated variable for BMI [weight (kg) / (height [m^2^])] was used to create the following four categories: underweight/healthy weight (<25.0), overweight (25.0 to <30.0), obesity I (30.0 to <35.0), and obesity II (≥35.0).

^†††^ The health care access module was optional; five, eight, 11, and seven U.S. states and territories contributed data in 2017, 2018, 2019, and 2020, respectively. In 2021, the following responses were harmonized with 2017–2020 health insurance/health care categories: 1) Medicare and Medigap (private health insurance plans sold to supplement Medicare), 2) Medicaid and state-sponsored programs, 3) other government programs and Indian Health Service. Persons reporting that Children’s Health Insurance Program was their primary source of health care insurance or who did not know or refused were excluded from analysis.

^§§§^ Statistically significant difference (p≤0.05) for health insurance type among male veterans. Employer- or union-sponsored is the reference group.

^¶¶¶^ Statistically significant difference (p≤0.05) for health insurance type among female veterans. Employer- or union-sponsored is the reference group.

**** Estimates are not included because they might be unreliable when the number of respondents is <50 or absolute CI width is >30%.

^††††^ The sexual orientation and gender identity module was optional; 28, 29, 31, 33, and 32 U.S. states and territories contributed data in 2017, 2018, 2019, 2020, and 2021, respectively.

^§§§§^ In 2017, sexual orientation was assessed using the question, “Do you consider yourself to be…” with the following response options: straight, lesbian or gay, bisexual, other, and don’t know/not sure. During 2018–2021, sexual orientation was assessed using the question, “Which of the following best represents how you think of yourself?” and response options: gay, straight (that is, not gay), bisexual, something else, and I don’t know.

^¶¶¶¶^ Self-rated health was assessed using the question, “Would you say that in general your health is” with the following response options: excellent, very good, good, fair, and poor. These were then combined into the following three categories: excellent/very good, good, and fair/poor.

***** Persons were categorized as having a disability if they answered “yes” to any of the six questions assessing the following disability types: vision, hearing, cognitive, mobility, self-care, or independent living.

Among men with disabilities, prevalences of arthritis were higher among veterans for all six disability types (hearing, vision, cognitive, mobility, self-care, and independent living) than among nonveterans (p<0.001). Among women with a disability, veterans had higher prevalences of arthritis among five of six disability types (all except self-care [p = 0.07]) than nonveterans (p<0.001). Age-adjusted arthritis prevalences among veterans with employer or union-sponsored health insurance (women = 23.5%; men = 21.2%) were significantly lower than those among veterans with Medicare and Medigap (private insurance plans sold to supplement Medicare) (women = 37.0%; men = 38.1%), Medicaid/other state-sponsored insurance (women = 39.9%; men = 27.5%), and TRICARE/Veterans Health Administration/Military insurance (women = 34.7%; men = 31.1% [p<0.001]).

Geographically, the age-adjusted prevalence of arthritis among male veterans ranged from 18.1% in DC to 35.8% in West Virginia (male veteran state median = 25.5%) (Table 2). The age-adjusted prevalence of arthritis among female veterans ranged from 21.8% in Hawaii to 39.3% in Arkansas (female veteran state median = 31.2%). Generally, the highest age-adjusted arthritis prevalence quartile among veterans, for both men and women, includes U.S. states in the southern and Appalachian regions (Figure). Eight states were in the highest quartile for state-specific arthritis prevalence among male and female veterans (Alabama, Arkansas, Kentucky, Michigan, Oklahoma, Rhode Island, Tennessee, and West Virginia).

**TABLE 2:** Jurisdiction-specific age-standardized* estimated prevalence of diagnosed arthritis^†^ among veterans,^§^ by sex — Behavioral Risk Factor Surveillance System, United States, 2017–2021

State	Men		Women	
	Estimated no.^¶^	Age-standardized % (95% CI)	Estimated no.^¶^	Age-standardized % (95% CI)
Alabama	174,000	31.0 (28.6–33.4)	21,000	34.2 (29.8–38.9)
Alaska	23,000	24.8 (22.4–27.5)	4,000	28.4 (23.0–34.6)
Arizona	198,000	25.0 (23.2–27.0)	25,000	30.7 (26.1–35.7)
Arkansas	92,000	27.1 (24.3–30.1)	11,000	39.3 (32.0–47.2)
California	667,000	22.8 (20.7–25.1)	66,000	23.3 (18.3–29.2)
Colorado	138,000	23.6 (22.1–25.1)	17,000	28.4 (24.8–32.3)
Connecticut	75,000	22.7 (20.5–25.0)	6,000	25.9 (20.8–31.9)
Delaware	26,000	21.8 (19.1–24.7)	4,000	28.0 (22.3–34.5)
District of Columbia	7,000	18.1 (15.9–20.4)	1,000	24.0 (18.4–30.7)
Florida	530,000	24.7 (22.5–26.9)	69,000	32.8 (28.0–37.9)
Georgia	275,000	26.4 (24.4–28.6)	41,000	29.9 (25.8–34.4)
Hawaii	37,000	20.6 (19.0–22.4)	4,000	21.8 (18.0–26.1)
Idaho	48,000	23.8 (21.3–26.5)	6,000	32.6 (26.4–39.5)
Illinois	230,000	22.9 (20.3–25.8)	24,000	30.8 (24.1–38.5)
Indiana	170,000	26.0 (24.1–27.9)	16,000	30.3 (25.2–36.0)
Iowa	76,000	24.7 (23.0–26.5)	6,000	28.3 (22.8–34.6)
Kansas	74,000	25.6 (24.2–27.1)	8,000	33.3 (29.6–37.2)
Kentucky	127,000	30.5 (27.8–33.3)	14,000	33.7 (27.6–40.3)
Louisiana	110,000	25.5 (23.4–27.8)	13,000	31.8 (26.8–37.1)
Maine	48,000	28.9 (26.4–31.4)	5,000	32.8 (27.9–38.2)
Maryland	147,000	25.4 (23.7–27.1)	23,000	31.3 (27.6–35.3)
Massachusetts	131,000	27.9 (24.6–31.4)	13,000	29.0 (23.4–35.3)
Michigan	264,000	28.1 (25.8–30.4)	25,000	36.9 (31.0–43.3)
Minnesota	117,000	22.1 (20.6–23.7)	9,000	25.0 (21.5–28.8)
Mississippi	83,000	28.4 (25.6–31.4)	11,000	32.9 (26.7–39.8)
Missouri	181,000	25.8 (24.0–27.7)	22,000	35.4 (30.5–40.5)
Montana	38,000	27.3 (25.2–29.4)	4,000	31.2 (26.2–36.6)
Nebraska	48,000	23.6 (21.9–25.3)	5,000	32.4 (28.2–37.0)
Nevada	81,000	24.6 (20.9–28.7)	13,000	35.9 (28.0–44.7)
New Hampshire	40,000	24.5 (21.9–27.4)	3,000	25.6 (20.5–31.5)
New Jersey	117,000	23.1 (19.8–26.8)	12,000	29.2 (21.2–38.6)
New Mexico	54,000	25.0 (22.5–27.7)	7,000	34.8 (28.5–41.6)
New York	287,000	22.7 (20.9–24.6)	33,000	29.0 (24.9–33.4)
North Carolina	294,000	26.0 (24.0–28.1)	40,000	31.2 (26.2–36.8)
North Dakota	20,000	25.5 (23.4–27.8)	2,000	27.8 (22.4–34.0)
Ohio	314,000	27.8 (25.9–29.8)	32,000	31.9 (27.7–36.4)
Oklahoma	122,000	29.1 (26.9–31.4)	17,000	36.1 (31.1–41.3)
Oregon	121,000	25.8 (23.7–28.1)	15,000	30.4 (25.6–35.6)
Pennsylvania	331,000	25.9 (23.5–28.4)	39,000	35.5 (28.5–43.2)
Rhode Island	29,000	27.7 (24.3–31.3)	3,000	37.9 (31.2–45.2)
South Carolina	172,000	28.5 (26.2–30.8)	21,000	31.0 (27.0–35.3)
South Dakota	25,000	25.5 (22.5–28.8)	3,000	23.8 (18.7–29.7)
Tennessee	203,000	27.8 (25.4–30.4)	29,000	38.8 (32.6–45.4)
Texas	610,000	23.3 (21.2–25.5)	90,000	28.4 (24.5–32.6)
Utah	56,000	26.2 (24.4–28.2)	5,000	31.7 (26.7–37.2)
Vermont	17,000	23.1 (21.2–25.2)	2,000	27.6 (22.3–33.6)
Virginia	260,000	25.9 (24.4–27.5)	5,000	29.0 (26.0–32.2)
Washington	204,000	23.6 (22.2–25.0)	30,000	35.7 (31.8–39.7)
West Virginia	73,000	35.8 (33.1–38.5)	5,000	35.3 (29.0–42.2)
Wisconsin	146,000	24.4 (21.9–27.2)	13,000	32.2 (25.6–39.5)
Wyoming	17,000	25.6 (23.2–28.2)	2,000	24.0 (19.3–29.3)
**Median**	—	25.5	—	31.2
**U.S. territory**				
Guam	3,000	23.9 (21.4–26.7)	<1,000	16.2 (11.9–21.7)
Puerto Rico	27,000	19.4 (16.4–22.9)	4,000	29.3 (21.4–38.5)
U.S. Virgin Islands	<1,000	13.8 (6.2–27.9)	<1,000	25.0 (16.3–36.4)

* Age-standardized to the 2000 U.S. Census Bureau projected adult population, using three age groups: 18–44, 45–64, and ≥65 years. https://www.cdc.gov/nchs/data/statnt/statnt20.pdf

^†^ Responded “yes” to the question, “Have you ever been told by a doctor or other health professional that you had some form of arthritis, rheumatoid arthritis, gout, lupus, or fibromyalgia?”

^§^ Veterans were defined as respondents who answered “yes” to the question, “Have you ever served on active duty in the United States Armed Forces, either in the regular military or in a National Guard or military reserve unit?”

^¶^ Represents the estimated number of veterans with diagnosed arthritis, weighted to the noninstitutionalized U.S. civilian population using sampling weights provided in the Behavioral Risk Factor Surveillance System data.

**FIGURE:**
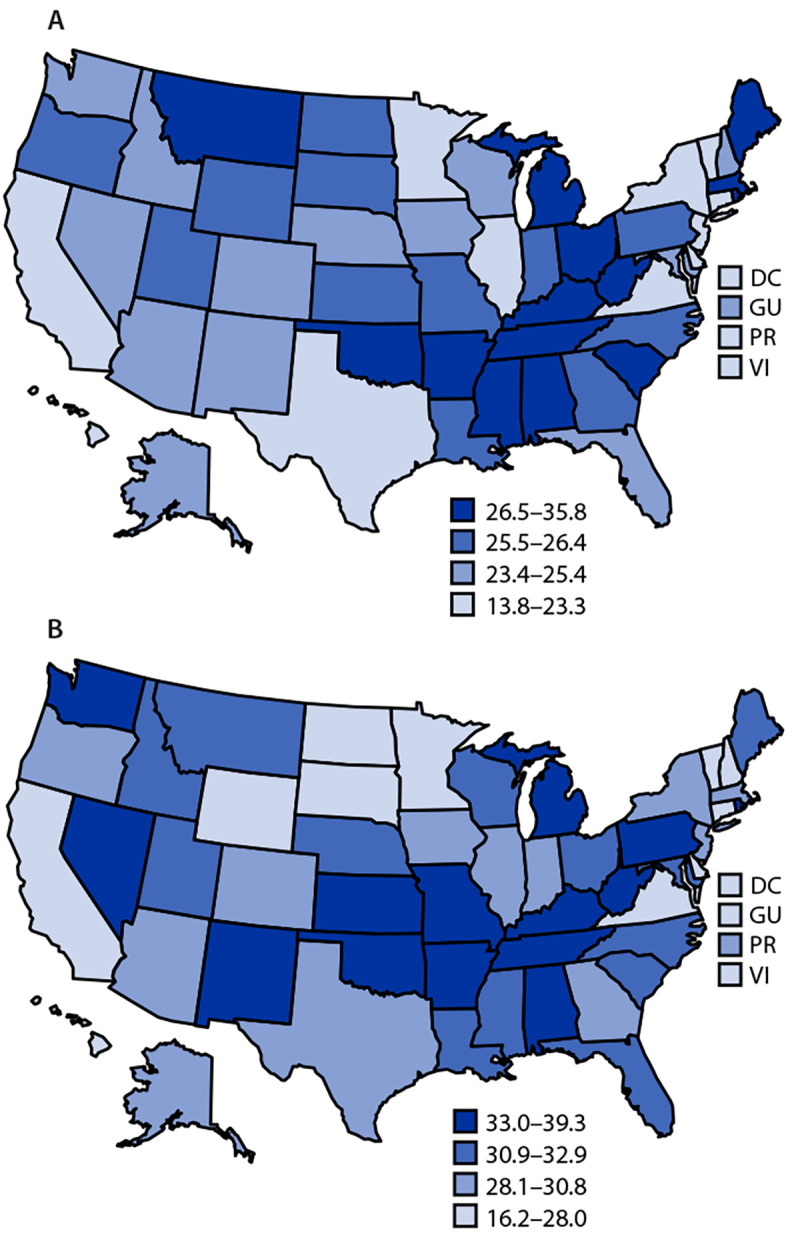
Jurisdiction-specific, age-standardized estimated arthritis prevalence (quartiles) among male veterans (A) and female veterans (B) — Behavioral Risk Factor Surveillance System, United States, 2017–2021 **Abbreviations:** DC = District of Columbia; GU = Guam; PR = Puerto Rico; VI = U.S. Virgin Islands.

## Discussion

In this study, approximately one third of veterans reported diagnosed arthritis. This report indicates that associations between sex, age, and disability status and arthritis prevalence reported for the general population (*3*) are also evident among veterans. Among veterans, the prevalence of arthritis was higher among women than men, and higher among veterans with disabilities than veterans without disabilities. Among men aged 18–44 years, the arthritis prevalence among veterans was double that among nonveterans, and among women aged 18–44 years, the arthritis prevalence among veterans was 60% higher than among nonveterans. This suggests that younger veterans might be living longer with arthritis and arthritis-attributable outcomes relative to nonveterans, which might result in higher rates and longer periods of work disability and lost wages (*6*,*7*). An analysis conducted using 2013 data estimated that among adults aged 18–64 years, adults with arthritis earned 9% ($3,361) less per year, compared with adults without arthritis (*6*). Therefore, younger veterans might be a prime population for prevention and interventions to help alleviate their symptoms and improve health outcomes. This report also describes geographic differences in arthritis prevalence among veterans, which can help to guide resource allocation and partnership development for the promotion or delivery of arthritis-appropriate interventions.

### Limitations

The findings in this report are subject to at least six limitations. First, BRFSS data are self-reported, which can result in recall and social desirability biases. Second, the data are cross-sectional; therefore, a causative relationship between military service and the development of arthritis cannot be inferred. Third, the BRFSS survey does not collect information on duration of military service or occupation type or activities while serving; therefore, arthritis prevalence across these characteristics could not be assessed. Fourth, these findings are not generalizable to U.S. adults without access to a landline or cell phone (e.g., persons experiencing homelessness or incarceration). Fifth, the current analyses did not examine confounding effects related to underlying differences in the distribution of age, sex, or race and ethnicity within the veteran population; future analyses might benefit from multivariable effect modification analyses. Finally, low response rates for individual states could result in nonresponse bias; however, the application of sampling weights helps address this bias.

### Implications for Public Health Practice

Arthritis prevalence among veterans is higher than among nonveterans, especially among male and female veterans aged <45 years and those with disabilities, providing rationale for prioritizing these subgroups for secondary and tertiary prevention efforts. These efforts might include dissemination of CDC-recognized arthritis-appropriate evidence-based interventions (AAEBIs), which are no- or low-cost physical activity and chronic disease self-management programs offered through community-based settings known to improve arthritis outcomes (*8*). Although veterans of all ages might benefit from AAEBIs to manage arthritis symptoms, younger veterans might have longer years of life lived with arthritis-attributable pain or disability, and therefore might receive additional benefit from AAEBIs to prevent or delay disease progression, disability, and functional limitations that might occur over time.

State-specific age-standardized prevalences of arthritis among male and female veterans can be used to guide state-level partnership development and resource allocation for addressing arthritis among veterans. Multisectoral partnerships among public health departments, community-based organizations, veteran-serving organizations, health care providers and payors (e.g., the Department of Veterans Affairs [VA], Medicare, and Medicaid) might help achieve equitable access to AAEBIs for all veterans. As one of the largest integrated health care systems serving an estimated 9 million veterans per year (*9*), the VA is particularly well-positioned to reach veterans with arthritis to provide interventions that might prevent or limit progression of the disease.

## Footnotes

*
https://www.cdc.gov/brfss/about/index.htm

† Data collected in 2021 from the U.S. Virgin Islands were not included in this analysis. In 2019 and 2021, New Jersey and Florida, respectively, were unable to collect BRFSS data during enough months to meet the minimum requirements for inclusion in the annual aggregate data set. No statistically significant differences in arthritis prevalence among veterans by survey year were found, enabling the use of a 2017–2021 aggregated data set.

§ The median combined landline- and cell phone–weighted American Association for Public Opinion Research response rate formula #4 for 2017–2021 were the following ranges: 2017: 45.9% (30.6%–64.1%); 2018: 49.9% (38.8%–67.2%); 2019: 49.4% (37.3–73.1); 2020: 47.9% (34.5%–67.2%); and 2021: 44.0% (23.5%–60.5%). https://www.cdc.gov/brfss/annual_data/annual_data.htm

¶ The overall sample size represents all persons with complete data for both the veteran and arthritis questions. Sample size for subgroup analyses might be smaller, because data for the items assessing the subgroup were missing.

** Age-standardized to the 2000 projected U.S. Census Bureau population with three age groups (18–44, 45–64, and ≥65 years). https://www.cdc.gov/nchs/data/statnt/statnt20.pdf

†† The sexual orientation and gender identity module was optional; 28, 29, 31, 33, and 32 U.S. states and territories contributed data in 2017, 2018, 2019, 2020, and 2021, respectively.

§§ In 2017, respondents were asked the question, “Do you consider yourself to be …” with the following response options: straight, lesbian or gay, bisexual, other, and don’t know/not sure. During 2018–2021, sexual orientation was assessed using the question, “Which of the following best represents how you think of yourself?” with the following response options: gay, straight (that is, not gay), bisexual, something else, and I don’t know. Proportions for the response categories were similar across years.

¶¶ Persons self-identifying as non-Hispanic American Indian or Alaska Native, Asian, Native Hawaiian or other Pacific Islander, other race, or multiracial were combined into “other, non-Hispanic.”

*** Responses to the question, “What is the highest grade or year of school you completed?” were combined into the following groups: 1) less than high school graduate: never attended school or attended only kindergarten, grades 1–8, or grades 9–11; 2) high school graduate or equivalent: grade 12 or general educational development certificate; 3) technical school degree or some college: college 1–3 years; and 4) college degree or more: college ≥4 years.

††† The calculated variables for income were harmonized across years to create four groups. For 2021 data, the following responses were combined into the “≥$50,000” category: $50,000 to <$100,000, $100,000 to <$200,000, and ≥$200,000.

§§§ The health care access module was optional; five, eight, 11, and seven U.S. states and territories contributed data in 2017, 2018, 2019, and 2020, respectively. In 2021, the following responses were harmonized with 2017–2020 health insurance/health care categories: 1) Medicare and Medigap, 2) Medicaid and state-sponsored programs, and 3) other government programs and Indian Health Service. Persons reporting that Children’s Health Insurance Program was their primary source of health care insurance or who did not know or refused were excluded from analysis.

¶¶¶ The calculated variable for body mass index [weight (kg) / (height [m^2^])] was used to create the following four categories: underweight/healthy weight (<25.0), overweight (25.0 to <30.0), obesity I (30 to <35.0), and obesity II (≥35.0).

**** Responses to the question, “Would you say that in general your health is…” were combined into the following categories: 1) excellent/very good, 2) good, and 3) fair/poor.

†††† Respondents were categorized as having a disability if they answered “yes” to any of the six questions assessing the following disability types: vision, hearing, cognitive, mobility, self-care, or independent living.

§§§§ 45 C.F.R. part 46.102(l)(2), 21 C.F.R. part 56; 42 U.S.C. Sect. 241(d); 5 U.S.C. Sect. 552a; 44 U.S.C. Sect. 3501 et seq.
